# Assembly of viral genomes from metagenomes

**DOI:** 10.3389/fmicb.2014.00714

**Published:** 2014-12-18

**Authors:** Saskia L. Smits, Rogier Bodewes, Aritz Ruiz-Gonzalez, Wolfgang Baumgärtner, Marion P. Koopmans, Albert D. M. E. Osterhaus, Anita C. Schürch

**Affiliations:** ^1^Department of Viroscience, Erasmus Medical CenterRotterdam, Netherlands; ^2^Viroclinics BiosciencesRotterdam, Netherlands; ^3^Department of Zoology and Animal Cell Biology, University of the Basque Country (UPV/EHU)Vitoria-Gasteiz, Spain; ^4^Systematics, Biogeography and Population Dynamics Research Group, Lascaray Research Center, University of the Basque Country (UPV/EHU)Vitoria-Gasteiz, Spain; ^5^Conservation Genetics Laboratory, National Institute for Environmental Protection and Research (ISPRA)Bologna, Italy; ^6^Department of Pathology, University of Veterinary Medicine HannoverHannover, Germany; ^7^Centre for Infectious Diseases Research, Diagnostics and Screening, National Institute for Public Health and the EnvironmentBilthoven, Netherlands; ^8^Center for Infection Medicine and Zoonoses ResearchHannover, Germany

**Keywords:** virus, pathogen, metagenome, virome, virus discovery, assembly, viral metagenomics

## Abstract

Viral infections remain a serious global health issue. Metagenomic approaches are increasingly used in the detection of novel viral pathogens but also to generate complete genomes of uncultivated viruses. *In silico* identification of complete viral genomes from sequence data would allow rapid phylogenetic characterization of these new viruses. Often, however, complete viral genomes are not recovered, but rather several distinct contigs derived from a single entity are, some of which have no sequence homology to any known proteins. *De novo* assembly of single viruses from a metagenome is challenging, not only because of the lack of a reference genome, but also because of intrapopulation variation and uneven or insufficient coverage. Here we explored different assembly algorithms, remote homology searches, genome-specific sequence motifs, k-mer frequency ranking, and coverage profile binning to detect and obtain viral target genomes from metagenomes. All methods were tested on 454-generated sequencing datasets containing three recently described RNA viruses with a relatively large genome which were divergent to previously known viruses from the viral families *Rhabdoviridae* and *Coronaviridae*. Depending on specific characteristics of the target virus and the metagenomic community, different assembly and *in silico* gap closure strategies were successful in obtaining near complete viral genomes.

## Introduction

Human and animal populations are continuously confronted with emerging viral infections (Delwart, 2007; Lipkin, 2010; Smits and Osterhaus, 2013). In a proportion of patients and animals suffering from disease, no pathogens can be detected using a range of sensitive diagnostic assays, suggesting the presence of unidentified viruses in human and animal populations (Bloch and Glaser, 2007; Denno et al., 2012). Classically, new viruses were identified by standard molecular detection methods, virus replication in tissue culture or animal experiments. Nowadays, in order to discover and characterize new or (re-) emerging viruses, metagenome sequencing is increasingly being used to identify viral pathogens. In addition, these techniques are more and more often being used to generate complete genomes of uncultivated viruses, but also other organisms (Delwart, 2007; Lipkin, 2010; Iverson et al., 2012; Albertsen et al., 2013; Smits and Osterhaus, 2013; Handley et al., 2014).

Metagenomic strategies to virus discovery rely on sequence-independent amplification of nucleic acids combined with next generation sequencing platforms instead of targeting specific genomic loci, thereby generating DNA sequences (i.e., reads) that align to various genomic locations for the numerous genomes present in the sample, including non-microbes (Sharpton, 2014). Common random amplification methods are multiple displacement amplification (MDA) or sequence-independent single-primer amplification (SISPA) (Hutchison et al., 2005; Spits et al., 2006; Delwart, 2007; Djikeng et al., 2008; Lipkin, 2010; Smits and Osterhaus, 2013). The advantages of sequence-independent amplification are simplicity and relative speed and the ability to identify and sequence hundreds of viruses simultaneously thereby allowing detection of new or previously unrecognized viruses that are highly divergent from already described ones (Bodewes et al., 2014a,c). Inherent to the approach is that a large fraction of the metagenome consists of sequences of other organisms than the viral targets, including host sequences, archaea, bacteria, and bacteriophages, despite physical enrichment strategies for virus particles that are often applied (Van Leeuwen et al., 2010; Kostic et al., 2012; Van Den Brand et al., 2012; Wylie et al., 2012; Bodewes et al., 2013; Schurch et al., 2014).

Metagenomic sequence data analysis with the aim to identify viral sequences presents several challenges. Datasets are relatively complex and large. In addition, the obtained viral reads in metagenomes can either originate from taxonomically informative genomic regions and even provide insight in the biological function of the encoded protein or originate from less conserved genomic regions for which biological functions are difficult to assign. Current strategies rely mostly on filtering steps to remove host nucleic acid from metagenomes either before or after sequencing and analysis of the data, including assembly and homology searches against annotated sequences in public databases (Woyke et al., 2006; Chew and Holmes, 2009; Schmieder and Edwards, 2012; Garcia-Garcera et al., 2013; Prachayangprecha et al., 2014; Schurch et al., 2014). Untargeted metagenomic approaches have enabled the identification of numerous newly emerging or previously unidentified viral pathogens in recent years. However, obtaining full-length viral genomes from metagenomic datasets remains challenging.

The number of reads obtained from a specific virus in metagenome samples is correlated to the viral load in the sample under study (De Vries et al., 2012; Prachayangprecha et al., 2014). In some cases, the number of reads in the sample is sufficient to permit enough read overlaps to establish longer contiguous sequences (contigs). However, direct assembly of complete viral and bacterial genomes from metagenomic data can involve a large amount of manual curation (Handley et al., 2014; Sharpton, 2014) as most pathogen genomes are not completely represented by reads and most viral communities are highly diverse (Mavromatis et al., 2007; Mende et al., 2012). Currently, full-length viral genomes are often obtained with additional experimental approaches based on PCR amplification with specific primers designed on obtained reads or contigs and/or 5′ and 3′ RACE PCR in combination with a Sanger sequencing approach (Van Leeuwen et al., 2010; Siegers et al., 2014). However, by optimally mining sequences in metagenomes, the likelihood and speed of identifying viral reads and the level of viral genome completeness can be increased and the need for laboratory follow-up minimized. In the present study, we describe and compare methods to obtain viral target genomes from metagenomes using a retrospective approach on 454-sequencing datasets containing three recently described viruses from the families *Rhabdo*- and *Coronaviridae*.

## Methods

### Datasets

The first metagenome dataset was obtained from a cell culture supernatant (CCS) containing a rhabdovirus-like virus isolated from tissue collected from a stranded white-beaked dolphin (*Lagenorhynchus albirostris*) (Osterhaus et al., 1993; Siegers et al., 2014). Genetic and phylogenetic characterization of the dolphin rhabdovirus (DRV) revealed that it was closely related to rhabdoviruses of the genera *Perhabdovirus* and *Vesiculovirus* found in fish (Siegers et al., 2014). In the second case, a highly divergent rhabdovirus, called red fox fecal rhabdovirus (RFFRV) was identified during a metagenomic survey of feces of red foxes from Spain (*Vulpes vulpes*) (Bodewes et al., 2014c). The last metagenome dataset was from lung tissue of a dead Indian python (*Python molurus*) with pneumonia, in which a novel nidovirus belonging to the family *Coronaviridae* within the order *Nidovirales* was identified. It was the first description of a reptile nidovirus (python nidovirus, PNV) and phylogenetic analysis placed this virus in the subfamily *Torovirinae* (Bodewes et al., 2014a). These datasets were acquired using a random sequence amplification and deep sequencing approach on a 454 GS Junior instrument (Roche) as previously described by Van Leeuwen et al. (2010), Bodewes et al. (2013, 2014a,c). At present full-length genomes (DRV) or expected complete coding sequences (PNV, RFFRV) are available.

### Assembly methods

Four different assembly methods, exhaustive iterative assembly (Schurch et al., 2014), CLC Genomics Workbench 6.0.4 assembler (CLC bio, Aarhus, Denmark), Genovo version 0.4 (Laserson et al., 2011), and Newbler 2.5 (Roche), were compared in their efficiency of detecting viral reads in the three metagenome datasets. The originally used method was iterative exhaustive assembly. Iterative exhaustive assembly of sequences is part of a virus discovery pipeline written in the python programming language (Python 2.7) that includes trimming of reads and initial assembly with Newbler (454GS Assembler version 2.7, Roche), with standard parameters. Trimmed reads and initial contigs were subjected to assembly by CAP3 (VersionDate: 12/21/07) (Huang and Madan, 1999) with standard parameters. The resulting singletons and contigs were iteratively assembled by CAP3 until no new contigs were formed.

Subsequently, the trimmed reads were mapped back to the identified taxonomic units with Newbler (454 GSMapper version 2.7, Roche) with standard parameters (Schurch et al., 2014). CLC Genomics Workbench 6.0.4 assembler (CLC bio, Aarhus, Denmark) was run with the previously trimmed reads with automatic bubble and word size. Genovo version 0.4 was run with 40 iterations and otherwise default values (Laserson et al., 2011). Newbler 2.5 (Roche) was run with default values.

### Determination of taxonomic content

Contigs and singletons of the iterative assembly approach that were longer than 75 bases were filtered with Dustmasker which is part of the NCBI-BLAST+ 2.2.25 suite of tools for sequences that contain more than 60% low complexity sequences (Camacho et al., 2009). After filtering of low complexity sequences, the remaining taxonomic units were subjected to a BLASTN search against a database that contained only nucleotide sequences from birds (Aves, taxonomic identifier 8782), carnivores (Carnivora, taxID 33554), primates (Primates, taxID 9443), rodents (Rodentia, taxID 9989), and ruminants (Ruminantia, taxID 9845) with an *e*-value cut-off of 0.001 for subtraction of potential host sequences. Sequences without hits in the host-BLAST were then subjected to a BLASTN search against the entire nt database with an *e*-value cut-off of 0.001. All sequences without hits were then subjected to a BLASTX search against protein sequences present in the nr database. BLAST hits were categorized by assigning a taxonomic category.

The percentage of viral reads in the sequence datasets and read coverage of the target genome using different assembly methods were determined by mapping trimmed reads to reference genomes with GSMapper Version 2.7 (Roche) with a minimum overlap identity of 80%.

### Remote homology search

All contigs were translated in six frames. Hidden Markov Models (HMMs) of PFAM families associated with *Rhabdoviridae* (pfam14314, pfam00945, pfam02484, pfam03216, pfam03342, pfam03012, pfam03397, pfam04785, pfam05554, pfam00922, pfam00974, pfam06326) were used to search the translated contigs of the metagenome datasets with rhabdoviruses with HMMER3.1 (Punta et al., 2012). Accordingly, HMMs of 45 PFAM families associated with *Coronaviridae* (pfam05213, pfam06460, pfam04694, pfam09408, pfam08717, pfam08716, pfam08715, pfam06478, pfam06471, pfam05409, pfam03262, pfam03053, pfam02723, pfam01601, pfam01600, pfam00937, pfam08779, pfam12383, pfam12379, pfam12133, pfam12124, pfam12093, pfam11963, pfam11633, pfam11501, pfam11395, pfam11289, pfam11030, pfam10943, pfam09401, pfam08710, pfam06336, pfam06145, pfam05528, pfam04753, pfam03905, pfam03622, pfam03620, pfam03617, pfam03187, pfam02398, pfam01635, pfam09399, pfam01831) were used to search the translated contigs of the PNV metagenome.

### Motif discovery and motif search

Motif sequence patterns were discovered with MEME Version 4.9.1 (Bailey et al., 2009) by allowing any number of repetitions on the sequence. The best scoring detected motif distributed over the seed contig was then used to search the motif in the collection of all contigs longer than 500 bases in all three datasets with MAST (Bailey et al., 2009) with an *e*-value lower than 0.1.

### Coverage profile binning

The average coverage of all contigs identified using exhaustive iterative assembly was calculated by dividing the number of reads covering the contigs by its length, as determined by the mapping procedure of the virus discovery pipeline. Frequency of binned coverage profiles was visualized in R statistical package version 3.1.

### K-mer frequency ranking

K-mer frequency was determined with the Bioconductor package biostrings (Pages et al., in press) for 3mers to 8mers for contigs larger than 1 kb in R statistical package version 3.1 (Team, 2012). Absolute differences between the k-mer frequencies of the seed contig and all other contigs were summed among different k-mer lengths and ranked, and visualized in relation to contig size.

### Accession numbers

Viral genome sequences used in this study were taken from Genbank, accession numbers KF958252 (DRV), KF823814 (RFFRV), and KJ935003 (PNV).

## Results

### Evaluation of different assembly algorithms

The objective of this study was to test and evaluate methods to increase the likelihood and speed of identifying viral reads and the level of viral genome completeness from metagenomic datasets generated on the 454-sequencing platform. The three 454-sequencing datasets obtained from a CCS, a red fox fecal (RFF) metagenome, and a tiger python lung tissue (TPLT) metagenome contained 69,358, 56,174, and 135,812 sequence reads, respectively (Table 1). These reads were analyzed with an automatic analysis pipeline that included stringent quality and length trimming, exhaustive iterative assembly, and low complexity filtering (Schurch et al., 2014). A total of 28,207, 32,455 and 50,024 reads from the CCS and the RFF and TPLT metagenome, respectively were subjected to homology searches (Table 2). The analysis showed a high variety among almost all taxonomic categories in the three different datasets (Table 2). The overall viral content determined by homology search was relatively low (0.72%) in dataset 3 (TPLT metagenome), and high (30.21 and 68.05%) in datasets 1 and 2 (CCS and RFF metagenome, Table 2).

**Table 1:** **Description of deep sequencing datasets**.

	**CCS**	**RFF**	**TPLT**
Total number of reads	69358	56174	135812
Assembled metagenome (%)	40.67	57.78	36.83
Reads identified by homology search as obtained from target virus (%)	27.67	5.82	0.11
Reads retrospectively obtained from target virus (%)	69.52	13.58	26.14

*Cell culture supernatant (CCS) containing Dolphin rhabdovirus (DRV), red fox feces (RFF) metagenome containing red fox fecal rhabdovirus (RFFRV) and python lung tissue (TPLT) metagenome containing python nidovirus (PNV)*.

**Table 2:** **Taxonomic composition of deep sequencing datasets**.

	**CCS**	**RFF**	**TPLT**
Unassigned	0.04	0.19	0.97
Virus	68.05	30.21	0.72
Unknown	3.90	10.35	49.39
Eukaryota	27.40	37.90	35.22
Bacteria	0.61	21.34	13.64
Archea	0	0	0.06

*Taxonomic composition per read in percentage of cell culture supernatant (CCS) containing Dolphin rhabdovirus (DRV), red fox feces (RFF) metagenome containing red fox fecal rhabdovirus (RFFRV) and python lung tissue (TPLT) metagenome containing python nidovirus (PNV). Unassigned, best BLAST hit without taxonomic assignment. Unknown, no homology to any database entry*.

Iterative exhaustive assembly resulted in assembled metagenomes containing between 40–60% of the original total amount of obtained reads (Table 1) of which the virome has a large dynamic range of reads depending on the sample under analysis. Unsurprisingly, the CCS dataset from an assumingly relatively pure virus culture supernatant had a high viral content, consisting predominantly of DRV. The viromes of the RFF metagenome showed a much smaller percentage of the RFFRV indicating the presence of multiple different viruses (Tables 1, 2).

**Figure 1:**
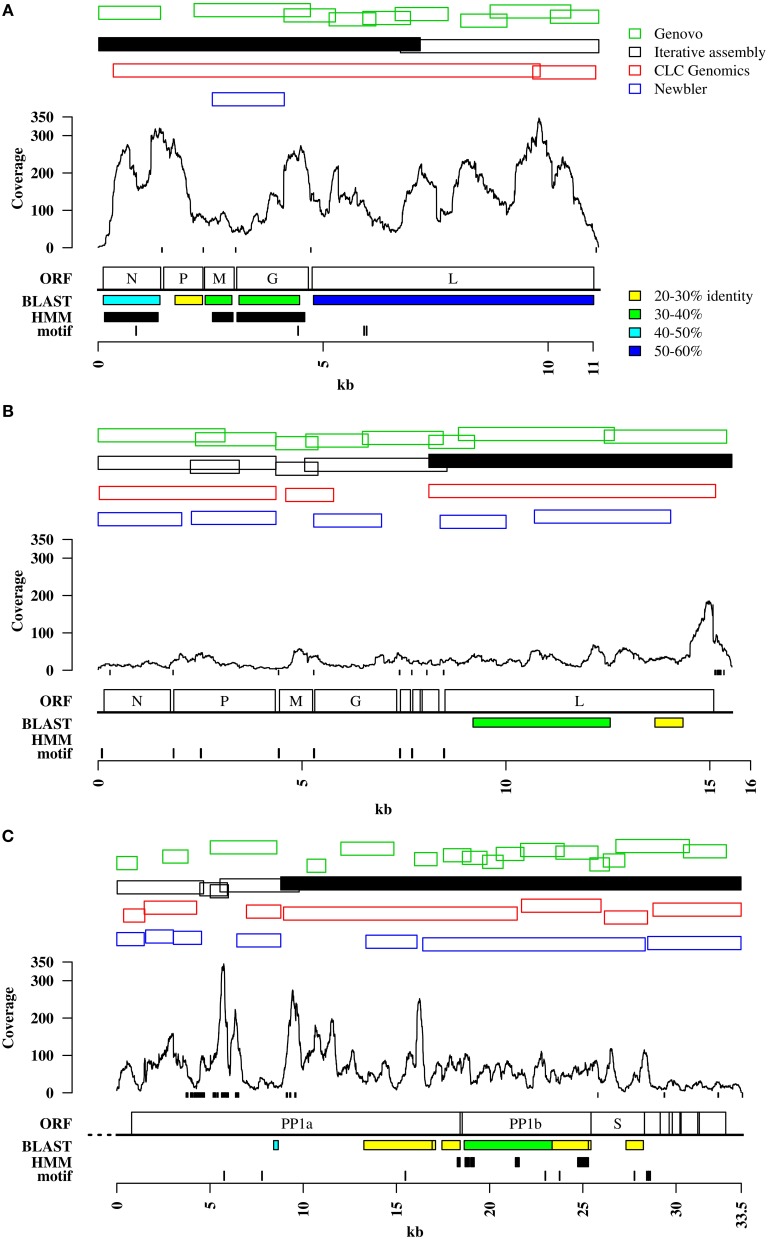
**Viral target genomes**. Panels **(A–C)** contain information on read coverage and contigs matching the viral genomes of DRV **(A)**, RFFRV **(B)**, and PNV **(C)**, produced by different assembly algorithms. Shown are only contigs larger than 1 kb. Green: Contigs assembled through Genovo as described in the methods. Black outlined: Contigs assembled through iterative assembly. Black solid: Seed contig. Red: Contigs assembled through CLC Genomics workbench assembler. Blue: Contigs assembled through Newbler assembler. Small black boxes at the bottom of the read coverage line mark stretches of low sequence complexity. “ORF” indicates the genome organization as described below. “Motif” shows the location of sequence motifs. Motifs are shown in detail in Figure S1. “BLAST” shows regions with sequence homology as determined by BLASTX. Colored boxes show sequence identity to the best BLAST hit as indicated on top. “HMM” indicates region with remote homology identified by PFAM profiles, if any. Ruler at the bottom indicates sequence lengths in kilobases. **(A)** DRV, Dolphin rhabdovirus; N, nucleoprotein; P, phosphoprotein; M, matrix protein; G, glycoprotein; L, large protein. **(B)** RFFRV, Red fox fecal rhabdovirus; N, nucleoprotein; P, phosphoprotein; M, matrix protein; G, glycoprotein; L, large protein; no abbrevation, alpha 1,2,3 protein. **(C)** PNV, Python nidovirus; PP1a, polyprotein 1a; PP1b, polyprotein1b; S, spike glycoprotein; no abbreviations, minor membrane protein, membrane protein, nucleocapsid protein, minor membrane protein 2, putative hemagglutinin-neuraminidase protein. Striped line at 5′ end indicates putative unresolved 5′ end.

Genomes of DRV, RFFRV, and PNV were not completely assembled by the exhaustive iterative assembly approach implemented in the automated analysis pipeline. The largest contigs were 7291 bases (64.32% of DRV, DRV seed contig) and 7682 bases (47.9% of RFFRV), respectively, of an expected size of 11 to 15 kb for *Rhabdoviridae* and 24,734 bases (73.68% of PNV) of an expected 30 kb for *Coronaviridae* (Figures 1A–C). Interestingly, retrospective mapping of reads to the viral target genomes showed that a large percentage of the sequences identified as “unknowns” by homology searches in the TPLT and RFF metagenome were actually derived from the target genome, most likely from parts of the target genomes without detectable similarity to any other viral protein in the BLAST database (Tables 1, 2).

To evaluate if other assembly algorithms would be able to directly assemble the complete target viral genomes from the deep sequencing data, we compared the contigs assembled from trimmed reads by iterative assembly, CLC Genomics Workbench assembler, Genovo and Newbler. While Genovo and Newbler both produced many small contigs covering part of the target genomes (Figures 1A–C), CLC Genomics Workbench assembler and the iterative assembly approach produced a similar number of contigs (two to five). However, large contigs (>1 kb) produced by iterative assembly covered the target genomes more completely than any other set of contigs obtained with other assembly algorithms. None of the assemblers tested here was able to completely assemble the viral genomes from the reads into a single contig (Figures 1A–C).

The contigs produced by iterative assembly and CLC Genomics Workbench for DRV were clearly overlapping (Figure 1A) and could be fused to a single assembly of a complete DRV genome by manual curation. For RFFRV in dataset 2, contigs assembled by iterative assembly and Genovo overlapped. However, a very small overlap of only five nucleotides between position 4356 and 4361, probably due to the combination of a drop in coverage and a stretch of sequence with low complexity (Figure 1B) did not allow us to retrieve a complete viral genome. Moreover, with the exception of the largest contig (the RFFRV seed contig), no other RFFRV contigs had a homolog in the NCBI nucleotide or protein database. The minor overlap, in combination with absence of homology, prevented assembly of a complete RFFRV genome. Similarly, the overlaps between contigs of PNV obtained with different assembly algorithms, in combination with the absence of homology, were insufficient to conclusively obtain a full-length PNV genome. In the TPLT metagenome, a 24.7 kb contig had a stretch with an average identity of 29% amino acid identity to the replicase polyprotein of Berne virus, subfamily *Torovirinae* (Figure 1C). This contig was used as seed contig. Overall, the data indicate that iterative exhaustive assembly seems to perform best in terms of production of large contigs and coverage of target genomes compared to other assemblers. Thus, for further analysis we used the set of contigs produced by iterative assembly. It is of note, however, that using a combination of different assembly algorithms may result in a higher level of completeness of target genomes if not complete genome assembly.

### Remote homology search

In absence of BLAST-detectable sequence homology to previously described viruses for some stretches of the target genomes we attempted to use methods that are able to detect remote homologs, i.e., profile hidden markov models (Figures 1A–C). To retrieve and link contigs of the viral target genomes we used profile HMMs of protein domains present in *Rhabdoviridae* and *Coronaviridae*, respectively. For *Rhabdoviridae*, 12 domains were present in PFAM, from nucleocapsid, spike and matrix proteins. Searching the translated contigs of the CCS containing DRV identified three regions (Vesiculovirus matrix protein—PF06326.7, Rhabdovirus nucleocapsid protein—PF00945.13 and the Rhabdovirus spike protein—PF00974.13) covered by several contigs (Figure 1A, contigs smaller than 1 kb not shown). The RFF metagenome did not give any hits (Figure 1B). For *Coronaviridae* in the TPLT metagenome, 44 HMMs were present in PFAM, again covering all proteins families. Three *Coronaviridae*-specific HMMs identified several contigs, the Coronavirus NSP13 (F06460.7), the RNA synthesis protein NSP10 (PF09401.5) and the Coronavirus RPol N-terminus (PF06478.8). However, in all three datasets, all identified domains were already identified in contigs with BLAST sequence homology to a closely related virus (Figures 1A–C). Identification of regions with remote homology to family-specific domains did therefore not result in acquisition of additional genomic regions that were not identified by the original iterative assembly method in combination with homology search by BLAST.

### Motif search

**Figure 2:**
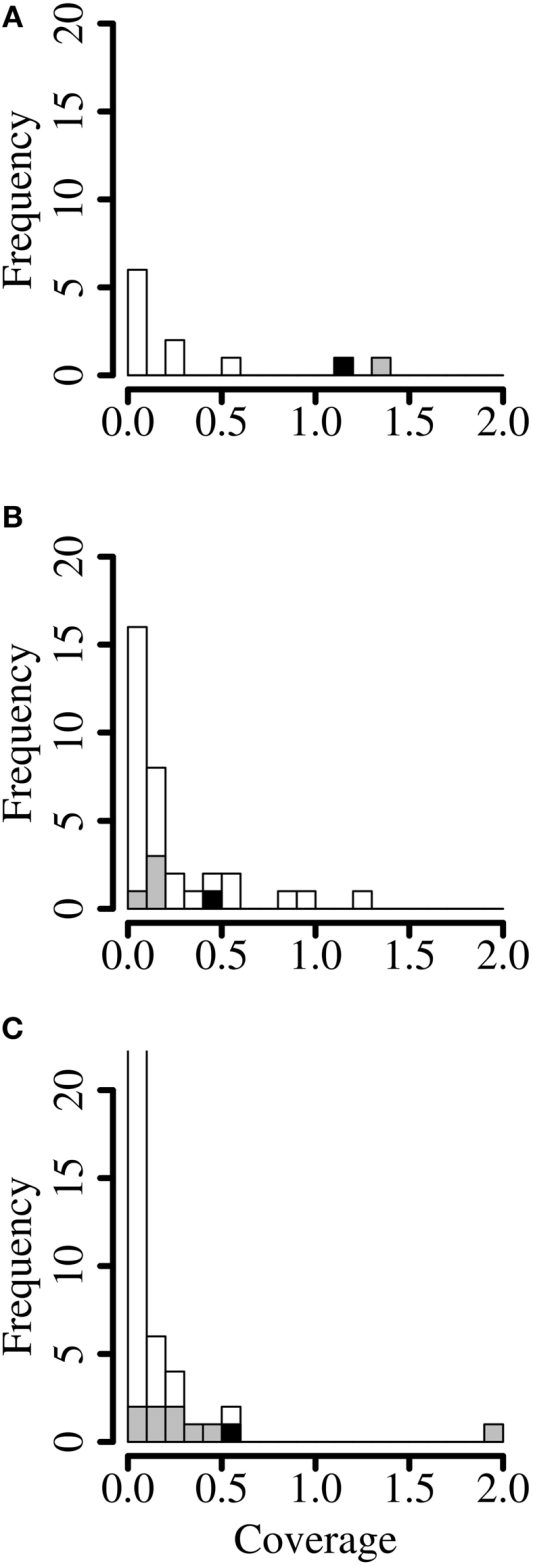
**Coverage profile binning**. Histograms of coverage (reads per base) of each contig of **(A)** of cell culture supernatant containing Dolphin rhabdovirus, **(B)** red fox feces containing red fox fecal rhabdovirus and **(C)** python lung tissue containing python nidovirus. Gray: contigs mapping to the finished viral genome. Black: seed contig. The first bar in the last panel is truncated for visibility (47%). Shown are only contigs larger than 1 kb.

A sequence motif is a DNA pattern that occurs repeatedly in a genome or in a group of related sequences. *De novo* motif discovery is independent of previously described motifs and their function. Motif discovery was performed on the seed contigs that showed homology to viruses of either the *Rhabdoviridae* or *Coronaviridae* family of metagenome 1 and 3 and on two adjacent, clearly overlapping RFFRV contigs of metagenome 2, including the RFFRV seed contig (Figure 2B). The highest scoring motif (Figures S1A–C) of each seed contig was then used to screen all available contigs of the deep sequencing datasets. Contigs were selected if they contained one or several occurrences of the motif at an *e*-value smaller than 0.01. Four additional DRV-matching contigs smaller than 1 kb (not indicated in Figure 1A) were identified in the CCS. In the RFF metagenome, four additional RFFRV contigs were identified and in the TLPT metagenome, one additional PNV contig exhibited the detected pattern (Figures 1B–C). No false positive contigs were identified with this method. Moreover, when the PNV motif was used to search all three viral genomes including the rhabdovirus genomes, it exhibited highest specificity for the PNV genome (*e*-value 4e-12), with *e*-values above 1 for RFFRV and DRV. The DRV and the RFFRV motif were most specific for their originating genome, while the *e*-value for the respective other rhabdovirus genome was relatively low (0.62 and 0.015), suggesting those motifs might be conserved among both rhabdovirus genomes. The high specificity of the motifs described here was also demonstrated when scanning all contigs of the three metagenomes: the motif discovered contigs of the respective viral genomes with a high specificity. The detection sensitivity however was restricted by the number of contigs that contained the motif. For example, the eight occurrences of the motif in the RFFRV genome were, with one exception, all found in intergenic regions. Contigs not containing intergenic regions could not be identified with this method. All occurrences of the detected motifs in the target genomes are indicated in Figures 1A–C. For both RFFRV and PNV, motif detection was able to identify contigs from genomic regions lacking BLAST or HMM-detectable homology. This information was sufficient to obtain the complete coding region of the RFFRV genome in combination with the iterative assembly approach.

### Coverage profile binning

In order to find additional contigs by coverage profile binning, the average coverage of every contig of the CCS and the two metagenomes was calculated by dividing the number of reads by the length of the contig. In the CCS dataset, an average coverage of 1.20 reads per base was achieved for the DRV seed contig. Accordingly, another contig that had a coverage of more than 1.1 read/base was obtained from the DRV genome (Figure 2A). All other contigs in the CCS dataset showed a lower coverage profile. In the RFF metagenome however, contigs with a similar coverage frequency as the seed contig of RFFRV (coverage of 0.42) were identified as putative plant genes, with a closest homolog to a hypothetical protein of *Medicago truncatula* (BLASTX *e*-value 6e-60, coverage of 0.45) or as part of the *Vulpes vulpes* mitochondrium (*e*-value 0.0, coverage of 0.58) (Figure 2B) and as two parts of a novel picobirnavirus, RFF picobirnavirus, isolate 40-2 (Bodewes et al., 2014b) (*e*-value 0.0, coverage of 0.299 and 0.99). In the TPLT metagenome, the seed contig of PNV was covered by 0.5 reads per base. Four of five contigs with a coverage profile of more than 0.2 were matching the PNV genome (Figure 2C), with the exception of one contig identified as hypothetical protein of *Clostridium thermocellum* (BLASTX *e*-value 4e-29). In conclusion, coverage profile binning identified additional contigs in two of the datasets.

### K-mer frequencies

**Figure 3:**
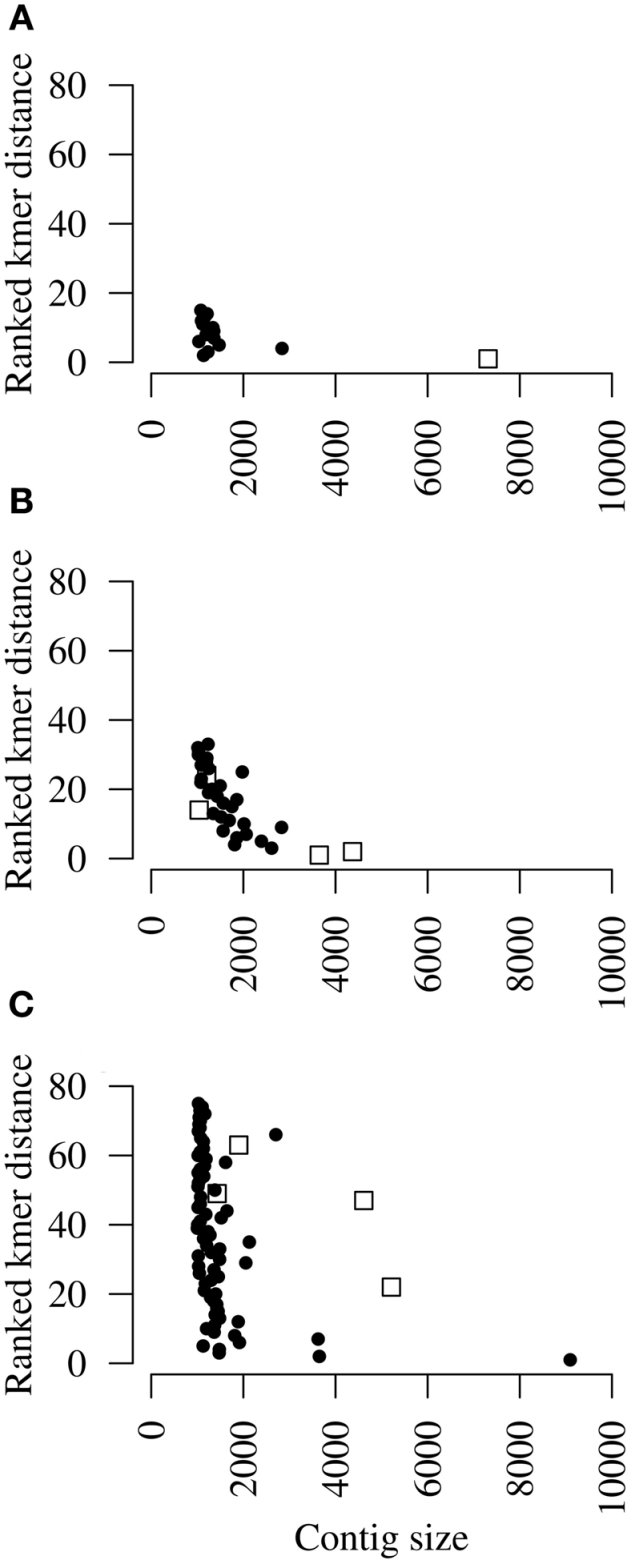
**K-mer profiling**. Dot plots showing ranked k-mer distance of each contig when compared to the k-mer profile of the seed contig of **(A)** Dolphin rhabdovirus (DRV), **(B)** red fox fecal rhabdovirus (RFFRV), and **(C)** python nidovirus (PNV) in relation to contig lengths. Open boxes indicate contigs that were retrospectively identified as originating from the target genomes. Shown are only contigs larger than 1 kb.

Another possible method to detect contigs that lack homology to known viruses but are part of the viral target genome is to determine k-mer frequencies. The frequency of every oligomer at length k was determined for *k* = 3, 4, 5, 6, 7 and 8 and ranked according to their absolute difference with the sum of the k-mer frequencies of the seed contig (Figure 3). Low ranking contigs have a similar k-mer frequency profile as the seed contig, whereas high ranking contigs differ in their k-mer frequency profile. For the CCS dataset, one 7.5 kb contig had a closely matching, high ranking k-mer frequency profile and was indeed originating from DRV (Figure 3A). Similarly, the two largest contigs (>3.5 kb) of RFFRV had the highest rank when compared to the frequency profile of the seed contig (Figure 3B). Two smaller contigs ranked at 14 and 24, suggesting that k-mer frequency profile clustering works better with long sequences. However, for PNV, the highest ranking, largest contig was obtained from the python host genome and contained among others a sequence for cytochrome C oxidase subunit (BLASTX *e*-value 0). Two large contigs matching the PNV genome ranked at 22 and 47, and two small contigs that were obtained from the PNV genome had an even higher rank (Figure 3C). While k-mer frequency ranking identified an additional part of DRV, and two large RFFRV contigs, all high-ranking contigs (rank 10 or less) of dataset 3 were unrelated to PNV.

## Discussion

We tested and compared different strategies to assist in identifying viral contigs and increasing the level of viral genome completeness from metagenomes. The retrospective nature of this study allowed us to compare the success of the strategies in retrieving three novel viral genomes from 454 metagenomic data. While different metagenome assembly strategies, especially for very large datasets of short read data, apply k-mer clustering or digital normalization and partitioning prior to assembly (Howe et al., 2014; Reddy et al., 2014), we here concentrated on strategies to link contigs after assembly *in silico*. Theoretically, these strategies can also be applied to contigs from metagenomes produced by other sequencing methods. A growing number of metagenome studies apply other next-generation sequencing techniques (e.g., Illumina), but 454 sequencing is still widely applied in viral metagenome studies, sometimes in combination with Illumina or PacBio sequences, because of the large read length (up to 800 bases) (De Vries et al., 2012; Grard et al., 2012; Philippe et al., 2013). In all three datasets presented here, the viral load of the samples was exceptionally high. Despite the high number of reads obtained from the target virus, direct assembly of the full genomes was not possible.

Metagenome assembly is a challenging task, because the number, nature, and abundance of the genomes present in the metagenome is unknown. Whole-genome assemblers are not suited for this task (Laserson et al., 2011; Peng et al., 2011; Lai et al., 2012; Namiki et al., 2012; Scholz et al., 2012). They assume even coverage and recognize high-coverage regions as repeats rather than a highly abundant species, or as an unevenly covered region introduced by amplification bias. Virus discovery relies heavily on low input material methods; therefore an amplification strategy is often necessary. Common random amplification methods, such as MDA or sequence-independent SISPA (Dean et al., 2002; Hutchison et al., 2005; Spits et al., 2006) are known to produce strong amplification biases leading to highly uneven coverage depths (Karlsson et al., 2013; Rosseel et al., 2013). Nevertheless the need for amplification makes these two methods still the most widely used in virus discovery (Allander et al., 2005; Djikeng et al., 2008; Hall et al., 2014). Introduction of amplification bias leads to stretches in the viral genome that are better covered than others. This can not only mislead assembly, it could also hamper detection of additional contigs by coverage profile binning. Accordingly, coverage binning was a successful strategy to link additional contigs for DRV and PNV, but not for RFFRV. Nevertheless, coverage profile binning was successfully applied to assemble viral genomes across a number of human gut metagenomes without the need of a reference (Nielsen et al., 2014) or to verify a cross-assembly of a novel bacteriophage in similar samples (Dutilh et al., 2014). Due to availability of a large amount of (fecal) sample for metagenome studies, amplification can often be avoided, which makes the application of coverage profile binning more straight-forward.

An additional issue in the case of viral metagenomes is the presence of distinct quasispecies sequences which can hamper direct assembly, especially at low sequencing depths. Using stringent assembly parameters that are necessary to avoid chimeras can lead to highly similar singletons or small contigs that are too diverse for assembly into a population sequence. This problem can be overcome by reference-guided assembly by a quasispecies assembler (Prosperi et al., 2013). However, this is currently not possible for divergent viruses which lack a reference genome. While many metagenome assemblers to date were designed to handle short-read data, only very few assemblers are dedicated to assembly of longer (i.e., 454) reads without any further information such as paired-end or mate-pair information. For this study, we used different assemblers, including an overlap-layout consensus algorithm (Newbler), an assembler that uses a generative probabilistic model (Genovo; Laserson et al., 2011), a de Bruijn graph algorithm (CLC Genomics Workbench), and an assembly strategy applying the combination of an OLC and a greedy algorithm (iterative assembly; Schurch et al., 2014). None of these strategies lead to a full reconstruction of the genomes of the novel viruses, but produced fragmented contigs. Overlaps between the contigs were often not recognized because of misassembled contig ends (not indicated in Figures 1A–C). These mis-assembled ends could represent chimeric contigs, i.e., assembled from reads from different species, but also chimeric reads due to chimera formation during PCR. Chimerism can not only prohibit successful assembly but can also lead to misclassification of the taxonomic content of the metagenome sample (Mavromatis et al., 2007; Pignatelli and Moya, 2011; Mende et al., 2012). Taxonomic “misclassification” of reads in the analysis described here, however, was rather due to the large number of taxonomic units without a homolog in the sequence databases. These reads were then classified as “unknowns.” Another challenge for recovery of viral genomes from metagenomes poses the segmented genomes of some viruses, with up to 12 segments for some viruses in the family *Reoviridae*, for example the Colorado tick fever virus (Attoui et al., 2000). Those segments can only be separately assembled, if possible, and need to be linked afterwards. The strategies described in this study can aid in identification of missing segments or contigs.

The strategy with the highest specificity was *de novo* motif discovery in the seed contig, and subsequent motif search in all contigs of the assembled metagenome. The (A/U)CU7 motif detected between open reading frames of RFFRV could serve as a transcription termination/polyadenylation sequence similar to other rhabdoviruses (Whelan et al., 2004).

Adjacent to this termination signal was a stretch of conserved nucleotides which might function as a transcription initiation signal. For the other detected motifs in DRV and PNV no obvious functions can be envisaged. However, their power to detect additional contigs matching the target genomes was only limited by the number of occurrences of the motif in the genome.

K-mer profile ranking detected large viral contigs with a similar profile as the seed contig in the CCS dataset and the RFF metagenome. In both cases, further manual curation or a laboratory follow-up would have been necessary to confirm the predictions made by this technique.

Assembly, in combination with motif discovery enabled retrieval of the complete RFFRV genome, with good results in k-mer frequency clustering. Two additional contigs of the PNV genome were identified by motif search, but linking of the remaining PNV contigs was only possible with frequency methods. Surprisingly, all methods applied here showed good results in retrieval of the full genome of DRV from the CCS dataset. This is most likely due to the high viral load which allowed assembly of the whole genome into two very long contigs in the first place. Therefore, we feel that the development of more efficient and dedicated metagenome assemblers, taking into account the specific characteristics of viral genomes, will lead to improved retrieval of viral pathogen genomes from metagenome sequences.

In conclusion, iterative exhaustive assembly, although highly stringent and thus excluding a large amount of data, is actually performing rather well compared to other assembly algorithms in that it covered the target genomes more completely than any other set of contigs obtained with other assembly algorithms. Nevertheless, the number of identified target virus reads and the level of viral genome completeness can be increased by combining data generated with different assembly algorithms. In addition, various methods can be applied to obtain additional genome fragments although a success rate cannot be predicted beforehand based on our analyses and probably depend largely on the dataset under study. These results indicate that a combination of these methods can be of great value to rapidly obtain additional genome information of a previously unknown virus.

## Author contributions

Rogier Bodewes and Anita C. Schürch conceived the study. Anita C. Schürch designed the experiments. Anita C. Schürch, Rogier Bodewes carried out the research. Saskia L. Smits contributed to the design of experiments. Anita C. Schürch prepared the first draft of the manuscript. Rogier Bodewes, Saskia L. Smits, Aritz Ruiz-Gonzalez, Wolfgang Baumgärtner, contributed materials. Saskia L. Smits, Marion P. Koopmans, Albert D. M. E. Osterhaus participated in the discussion and writing of the manuscript. All authors were involved in the revision of the draft manuscript and have agreed to the final content.

## Grant information

This work was partially funded by the Virgo Consortium, funded by the Dutch government project number FES0908, by Netherlands Genomics Initiative (NGI) project number 050-060-452 and ZonMW TOP project 91213058. A. Ruiz-Gonzalez holds a Post doc fellowship awarded by the Department of Education, Universities and Research of the Basque Government (Ref. DKR-2012-64) and was partially supported by the Research group on “Systematics, Biogeography and Population Dynamics” (Basque Government; Ref. IT317-10; GIC10/76).

### Conflict of interest statement

Drs. Albert D. M. E. Osterhaus and Saskia L. Smits are partly employed by Viroclinics Biosciences B.V., Rotterdam, Netherlands. The other authors declare that the research was conducted in the absence of any commercial or financial relationships that could be construed as a potential conflict of interest.

## Supplementary material

Figure S1 **(A)** Nucleotide sequence motif discovered in the Dolphin rhabdovirus (DRV). **(B)** Nucleotide sequence motif discovered in the red fox fecal rhabdovirus (RFFRV). **(C)** Nucleotide sequence motif discovered in the python nidovirus (PNV).
