# Supplementary figures and images for: Assembly of viral genomes from metagenomes
Source: Front Microbiol. 2014 Dec 18;5:714. doi: 10.3389/fmicb.2014.00714 (PMC4270193; doi:10.3389/fmicb.2014.00714)

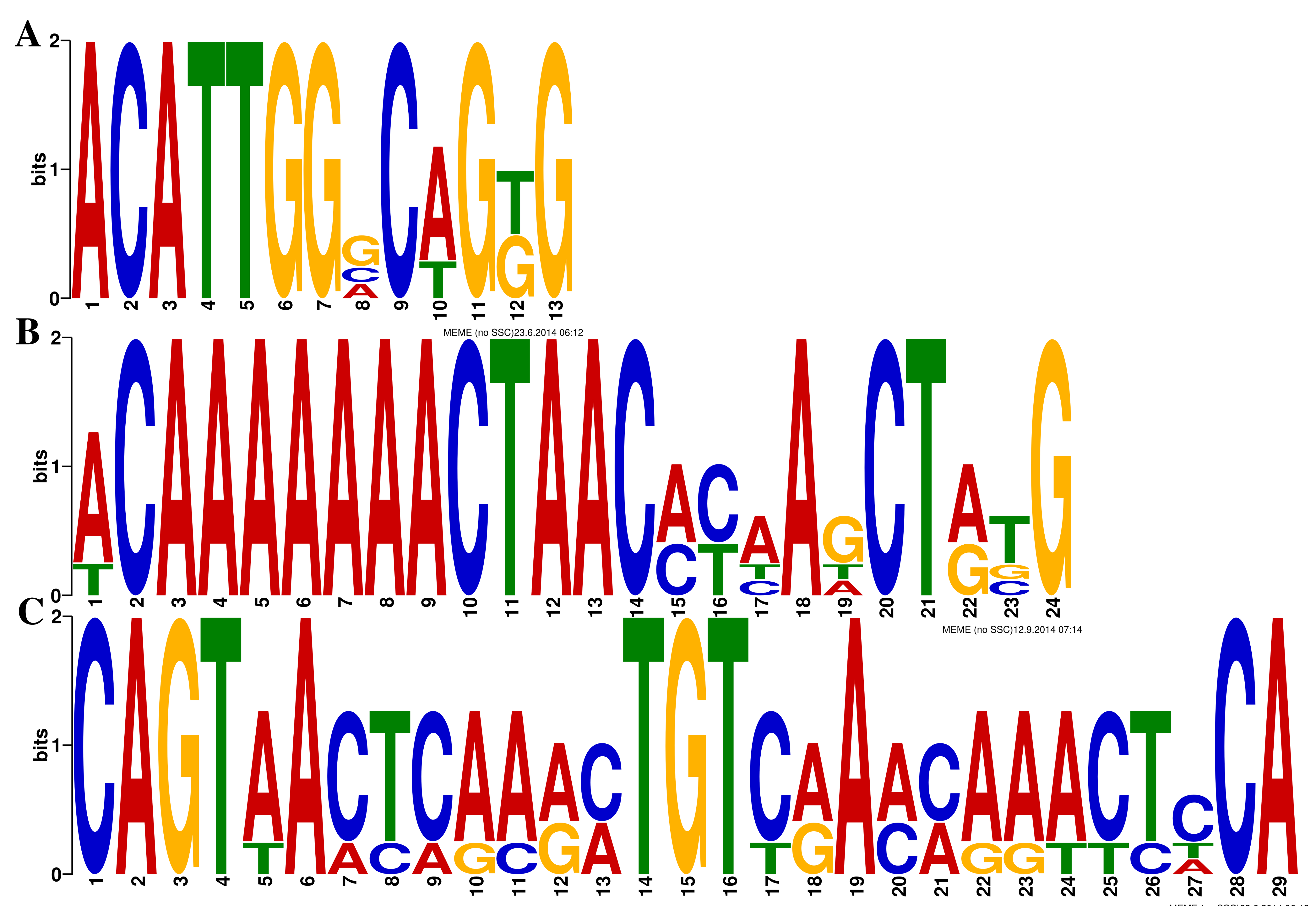

Supplement: Figure S1 — (A) Nucleotide sequence motif discovered in the Dolphin rhabdovirus (DRV). (B) Nucleotide sequence motif discovered in the red fox fecal rhabdovirus (RFFRV). (C) Nucleotide sequence motif discovered in the python nidovirus (PNV). [file Image1.JPEG]
